# Identification and Characterization of Process-Related Impurities of Trans-Resveratrol

**DOI:** 10.3797/scipharm.1301-17

**Published:** 2013-03-17

**Authors:** Balasubramanian Sivakumar, Raman Murugan, Annamalai Baskaran, Bhausaheb Pandharinath Khadangale, Saravanan Murugan, Udayampalayam Palanisamy Senthilkumar

**Affiliations:** Orchid Chemicals and Pharmaceutical Limited, Research and Development Centre, Sozhanganallur, Chennai 600 119, Tamilnadu, India.

**Keywords:** Resveratrol, Impurities, Structure characterization, HPLC, Structural elucidation, NMR, LC-MS

## Abstract

This article deals with the identification and characterization of process-related impurities of trans-resveratrol (3,5,4′-trihydroxystilbene), which exhibits several health benefits, including cancer prevention. During the synthesis of the bulk drug resveratrol, three new impurities were observed. The impurities were detected using the high-performance liquid chromatographic (HPLC) method, whose area percentages ranged from 0.05 to 0.3%. A systematic study was carried out to characterize them. These impurities were isolated by preparative HPLC and characterized by spectral data, subjected to co-injection in HPLC, and were found to be matching with the impurities present in the sample. LC-MS was performed to identify the mass of these impurities. Based on their spectral data (IR, NMR, and Mass), these impurities were characterized as 2-benzyl-5-[(*E*)-2-(4-hydroxyphenyl)ethenyl]benzene-1,3-diol [Impurity-B], 3-(benzyloxy)-5-[(*E*)-2-(4-hydroxyphenyl)ethenyl]phenol [Impurity-C], 5-{(*E*)-2-[4-(benzyloxy)phenyl]ethenyl}benzene-1,3-diol [Impurity-D). These compounds are not reported earlier as process-related impurities.

## Introduction

Resveratrol (3,5,4′-trihydroxystilbene) is a naturally-occurring phytoalexin produced by various plants in response to environmental stress or pathogenic attack. It is present in peanuts, mulberries, blueberries, and grapes and possesses numerous biological activities [1]. It was reported that resveratrol can prevent or slow the progression of a wide variety of illnesses, including cancer, cardiovascular disease, and ischaemic injuries, as well as enhance stress resistance and extend the lifespan of various organisms from yeast to vertebrates [2]. It is a strong antioxidant and free radical scavenger. Resveratrol antioxidant activity is essential in the prevention of chemical-induced cancer by inhibiting the initiation step of the carcinogenesis process, but it is also considered to inhibit cancer promotion and progression steps [3]. Trans*-*resveratrol (**1**) proved to be more active than its cis-isomer (**2**) in all of the reported bioassays [4]. *In vitro, ex vivo*, and animal experiments have shown that resveratrol possesses many biological attributes that favor protection against atherosclerosis, including antioxidant activity, modulation of hepatic apolipoprotein and lipid synthesis, and inhibition of platelet aggregation as well as the production of pro-atherogenic eicosanoids by human platelets and neutrophils. Red wine represents its main source in the human diet, and it has been proposed as a major constituent of the polyphenol fraction to which the health benefits of red wine consumption have been attributed [5]. It inhibited the development of preneoplastic lesions in carcinogen-treated mouse mammary glands in cultures and inhibited tumorigenesis in a mouse skin cancer model. These data suggest that resveratrol, a common constituent of the human diet, merits investigation as a potential cancer chemopreventive agent in humans [6].

**Fig. 1:**
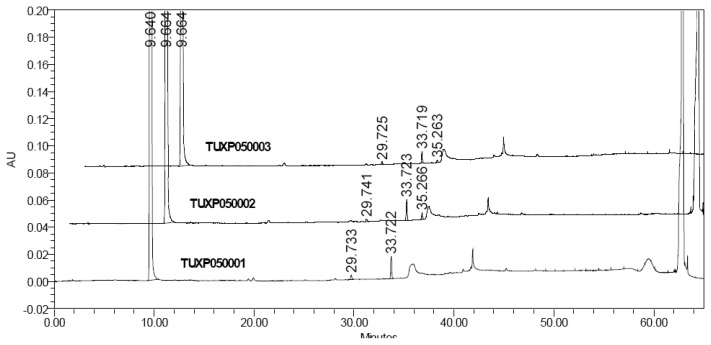
HPLC related substances data of trans-resveratrol samples.

During the analysis of laboratory batches of resveratrol, three impurities were observed by the HPLC method (Figure 1). In order to commercialize an API, it is a mandatory requirement by regulatory authorities to identify and characterize all the unknown impurities that are present at a level of more than 0.1% [7]. These impurities are required in pure form to check the HPLC method performance in areas such as specificity, linearity, range, accuracy, precision, limit of detection (LOD), limit of quantification (LOQ), robustness, system suitability testing, and relative response factor (RRF) [8]. These related substances are also used to check the accuracy of the analytical method of API.

**Fig. 2:**
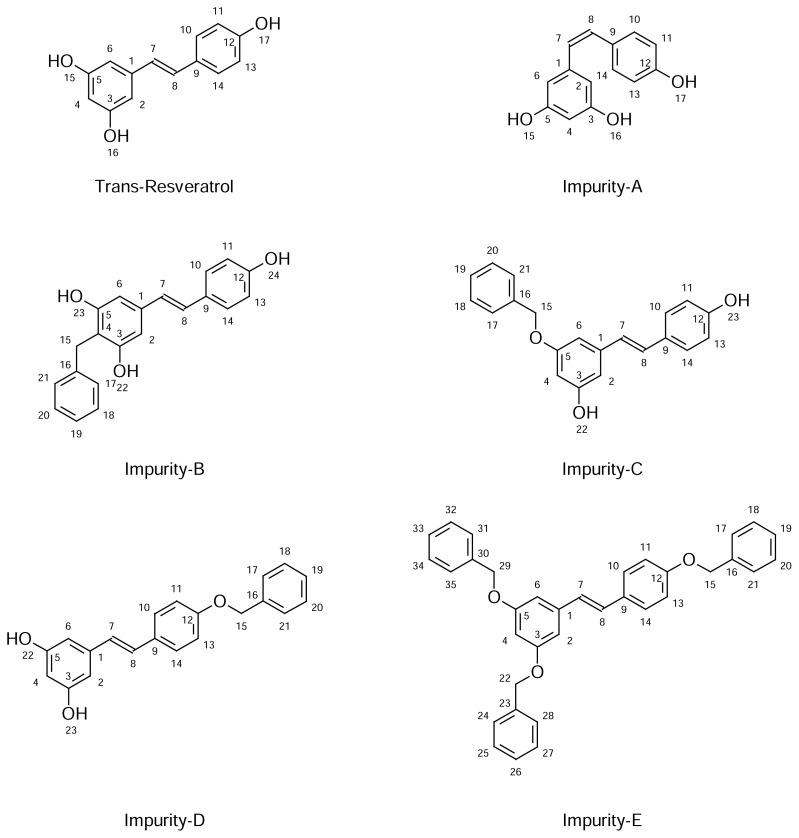
Structures of trans-resveratrol and its Impurities.

The structure of the three isolated impurities (Figure 2) related to the process is identified/characterized by the various characterization techniques such as UV, IR, NMR & Mass, and chromatographically by HPLC spiking studies. The Impurities **C** and **D** are reported in the literature as derivatives of resveratrol [9] and not reported as process-related impurities. Impurity-**B** is not reported in any of the earlier literature. Although cis-resveratrol (Impurity-**A**) and the penultimate intermediate (Impurity-**E**) in the manufacturing process were not observed in the samples, these two impurities were included in the study.

We have identified the following five impurities in the samples analyzed:

(*Z*)-5-[2-(4-Hydroxphenyl)ethenyl]benzene-1,3-diol [cis-resveratrol [Impurity-**A**],
2-Benzyl-5-[(*E*)-2-(4-hydroxyphenyl)ethenyl]benzene-1,3-diol [Impurity-**B**],
3-(Benzyloxy)-5-[(*E*)-2-(4-hydroxyphenyl)ethenyl]phenol [Impurity-**C**],
5-{(*E*)-2-[4-(Benzyloxy)phenyl]ethenyl}benzene-1,3-diol [Impurity-**D**);
1,3-(Dibenzyloxy)-5-[(E)-2-(4-benzyloxyphenyl)ethenyl)benzene [Impurity-**E**]

Resveratrol impurity-A (cis-resveratrol) was prepared by photoisomerization of trans-resveratrol as reported in the literature [4]. One of the impurities often found in low quality resveratrol is emodin which is not present in the samples analysed. There was no literature data on the process-related impurities other than on cis-resveratrol. The NMR spectral data of resveratrol and other related compounds were reported earlier [10–12].

## Experimental

### Samples and Chemicals

Samples of trans-resveratrol (Batch No. TUXP050001. TUXP050002, TUXP050003), were manufactured at Orchid Chemicals and Pharmaceuticals Ltd, Aurangabad, India. Three impurities were isolated by preparative HPLC and cis-resveratrol was synthesized in the laboratory and confirmed by identification by HPLC and LC-MS. HPLC grade acetonitrile was obtained from Merck, India. Water used for preparing the mobile phase was purified using the Millipore Milli-Q Plus (Milford, MA, USA) purification system. Chloroform-*d* and dimethylsulphoxide-*d6* were purchased from Euriso-top SA, France.

### Analytical HPLC

An in-house LC gradient method was developed for the analysis of trans-resveratrol and its impurities (Waters Alliance 2695 separations module & Waters 2487 Dual Absorbance Detector, with Empower software) using a Waters Symmetry C18 (250 × 4.6 mm), 5μ column at a flow rate of 1.5 mL/min. Column oven temperature was maintained at 30 °C. About 7.80 g of sodium dihydrogen orthophosphate dihydrate was dissolved in 1 L water and used as mobile phase A and acetonitrile was used as mobile phase B. The gradient compositions were employed as 0 to 10 min/20–30% B, 10 to 25 min/30–40% B, 25 to 35min/40–60% B, 35 to 40 min/60–80% B, 40 to 60 min/80% B, 60 to 65 min/80-20% B. Resveratrol and its related substances were monitored by a UV detector at 215 nm. There was an injection volume of 20 ul and injection delay of 10 min. About 50 mg of samples were dissolved in 1:1 of water and acetonitrile (100 ml) and then analyzed. This LC method was able to separate trans-resveratrol and its related substances.

### Preparative HPLC

Impurity isolation was carried using Waters 2000 Prep HPLC equipped with a UV detector monitored at 215 nm and a YMC C18, (250 × 30mm), 15 u column was used. The mobile phase used was water and acetonitrile in various ratios at a flow rate of 40 ml/min. The preparative HPLC fraction corresponding to the impurity was collected and confirmed by the analytical HPLC and further confirmed by mass using LC-MS. The multiple desired fractions were collected and the fractions were pooled together and lyophilized to get a pure solid. The purity of the isolated impurity was around 90%. The relative retention time of the isolated impurity was further confirmed by using HPLC and the molecular weight was confirmed by LC-MS.

### Liquid Chromatography Mass Spectrometry (LCMS)

LCMS experiments were carried out on a PE SCIEX API3000 LC-MS/MS equipped with a triple quadrupole analyzer, and the TurboIon spray sample introduction system. The LC instrument used for the LCMS experiments was the HPLC system with the Agilent 1100 G1311A(0) pump, Agilent 1100 G1329A(0) auto sampler, and Agilent 1100 DAD(0) detector. Analysis was carried out using the Waters Symmetry C18 (250 × 4.6 mm), 5μ column at 215 nm. Because of the non-volatile nature of the phosphate-buffer mobile phases used in the HPLC related substances method, the chromatographic system was modified with 10mM ammonium acetate at pH=4.5 as mobile phase A and 100 % acetonitrile was used as mobile phase B at a flow rate of 1.0 mL/min. Gradient elution was performed by using mobile phases A and B. Gradient compositions were employed as 0 min/30% B, 25 min/40% B, 35min/60% B, 40 min/80% B, 65 min/80% B.

### Mass spectrometry

The LC-MS analysis was performed on the API-3000 LC-MS/MS mass spectrometer [PE Sciex, Foster City, CA]. Initial LC-MS screening was performed by both positive and negative ion modes with the Turbo Ion Spray interface. No impurities were detected under the positive ion mode and all of the given resveratrol-related compounds were detected under the negative ion mode. The analysis was performed in the negative ionization mode and the following conditions: the ion source used with a voltage of −4200 V, declustering potential of 10 V, focusing potential of 90 V, entrance potential of 10 V. Nitrogen gas was used as the nebulizer gas at 60 psi. The molecular mass (m/z) of all of the impurities was observed as [M-H]^−^ in the negative ionization mode, where M is the mass of the impurity.

### NMR spectroscopy

The ^1^H NMR and ^13^C NMR experiments for resveratrol impurities were performed at 400.13 MHz and 100.62 MHz, respectively, on the Bruker Avance 400 MHz FT NMR spectrometer with a multinuclear BBO probe. DMSO-*d6* was used as the solvent. The ^1^H chemical shift values were reported on the δ scale in ppm, relative to TMS (δ = 0.0 ppm) and in the ^13^C NMR, the chemical shift values were reported relative to DMSO-*d*_6_ (δ = 39.50 ppm) as a reference. The DEPT-135 spectra revealed the presence of methyl and methine groups as positive peaks and methylene as negative peaks.

### IR & UV Spectroscopy

The IR spectra were recorded in the solid state as a KBr dispersion medium using the FT-IR (Perkin Elmer, Spectrum 65 & JASCO-FT-IR-430) spectrophotometer. The UV spectrum was recorded on a Shimadzu UV-visible spectrophotometer using acetonitrile as the medium.

## Results and Discussion

**Fig. 3:**
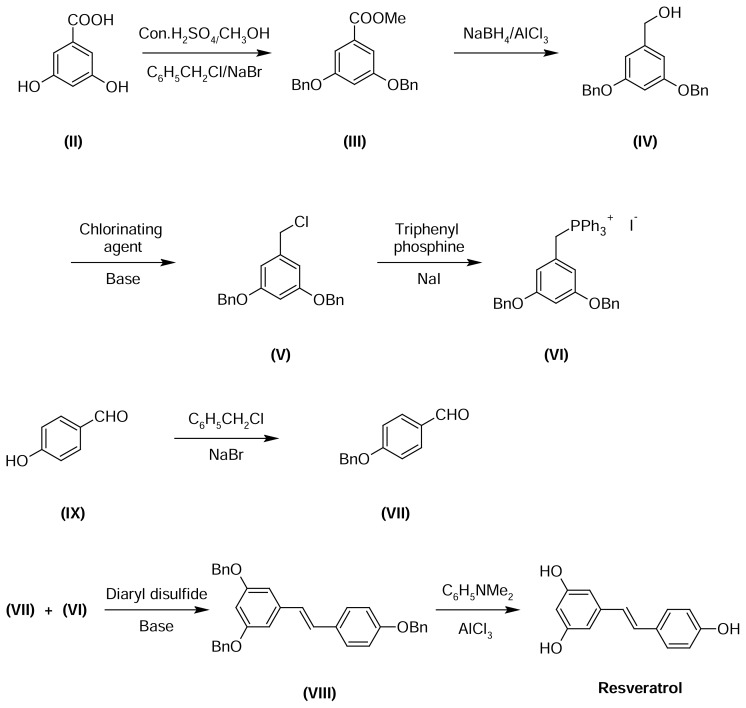
Scheme for trans-resveratrol Process

The trans-resveratrol manufacturing process [13] is shown in Figure 3, and the samples were analyzed by the HPLC method and three unknown impurities were observed at the level of 0.10% or more (Table 1). Cis-resveratrol (Impurity-**A**) and the penultimate intermediate (Impurity-**E**) used in the process were not observed. The unknown impurities were named as B, C, and D based on their relative retention times.

**Fig. 4:**
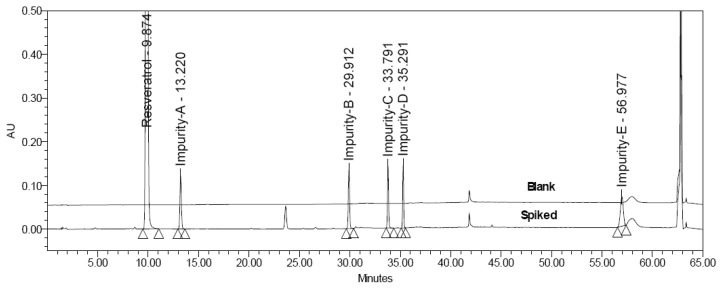
Overlaid chromatograms of blank and spiked impurities along with the sample

The three process-related impurities B, C, and D were isolated by preparative HPLC and confirmed by analytical HPLC and LCMS. An HPLC chromatogram obtained from a sample of trans-resveratrol spiked with all five impurities at the 0.15% level is shown in Figure 4. The structures of the impurities were confirmed by the NMR, LCMS, and FT-IR spectral techniques and discussed in the following sections. The ^1^H and ^13^C NMR assignments for trans-resveratrol and Impurities A, B, C, and D are shown in Table 2.

### Structural characterization of Impurity-A (cis-resveratrol)

The cis isomer of resveratrol was synthesized and the LCMS data confirm the molecular mass (m/z) of the impurity as 228.

^1^H NMR data indicate the alkene hydrogens at the C7 and C8 positions observed as two doublets at δ 6.25–6.35 ppm with a coupling constant J_1,3_ =12.28 Hz which confirms the cis orientation of the two alkene hydrogens [14]. In trans-resveratrol, these two alkene hydrogens were observed at around δ 6.79–6.90 ppm with a coupling constant J_1,3_ = 16.2 Hz. The three hydroxyl groups were observed as broad signals at δ 9.19 (2H’s) and 9.47 ppm, which disappear on D_2_O exchange studies. The aromatic hydrogen between the two hydroxyl substituents at the C4 position was found to be shielded to δ 6.05 ppm as a triplet with a meta coupling constant of J_1,3_ = 2.1 Hz, similar to that of trans-resveratrol. The hydrogens at the 2 and 6 positions were shielded to δ 6.11 and appear as doublets with a coupling constant J_1,3_ = 2.2 Hz, which is a characteristic coupling constant for the aromatic hydrogens in the meta position [14]. The remaining aromatic hydrogens at 10,11,13, and 14 appear as two doublets δ 6.60 & 7.06 ppm with a coupling constant of J_1,3_ = 6.7 Hz, which matches the coupling constant for the ortho coupling in aromatic systems, and also the splitting pattern matches the p-substituted aromatic compounds.

In the ^13^C NMR spectrum, three carbons deshielded more than the others and were observed as two sets at δ 156.7 and 158.4 ppm, which indicate that these carbons are attached to hydroxyl groups and assigned for the C3, C5, and C12 carbons. Due to the hydroxyl substituent effect at the ortho position, the carbons at C4, C6, C2, C11, and C13 appear more shielded at δ 101.6 (1C), 106.4 (2C), and 115.0 (2C) ppm. By using DEPT NMR data, all of the carbon signals were assigned. The NMR spectral data matches that reported for cis-resveratrol [4].

### Structural characterization of Impurity B

Impurity-B at RRT 3.03 was isolated by preparative HPLC. The LC-MS analysis of the fraction showed a molecular ion peak at m/z 317 which corresponds to the molecular weight of 316 as [M-H]^−^. The IR spectrum displayed characteristic absorptions at, 3422 (broad signal), 3027, 2929, 1511, and 1492 cm^−1^ which is indicative of O-H, aromatic C-H, methylene C-H, and aromatic –C=C stretching functionality. The structure was further supported by quaternary carbon signals, observed in the ^13^C spectrum and absent in the DEPT spectrum. ^13^C NMR accounted for 20 carbons and the DEPT spectrum displayed one negative carbon (CH_2_) signal and 13 positive carbon signals (11 aryl CH and 2 alkene CH) and the remaining seven extra signals that appeared in ^13^C are considered as seven quaternary carbons. ^1^H NMR results indicated that Impurity-B has five additional aromatic hydrogens and one more aliphatic hydrogen (-CH_2_-) when compared to that of trans-resveratrol. The above observation was also confirmed by ^13^C NMR and DEPT results.

The molecular mass of the impurity is 90 Da more than that of resveratrol. The benzyl group of molecular mass 91 Da seems to match that of the additional fragment. Based on the spectral data and the process [13] used for the synthesis of resveratrol, the impurity may be a benzylated one. The position of the benzyl group was confirmed by comparing the spectral data with that of trans-resveratrol, and the signal corresponds to the aromatic hydrogen at C4 at around δ 6.10 ppm in ^1^H NMR, and the aromatic carbon C4 at δ 101.9 ppm in ^13^C NMR was absent (Table 2). There were no significant changes in the other hydrogen and carbon signals. On the basis of ^1^H and ^13^C NMR data, the position of the benzyl group is located at the C4 position which is between the two hydroxyl substituents at C3 and C5.

From the above spectral studies, Impurity-B was confirmed as 2-Benzyl-5-[(*E*)-2-(4-hydroxy phenyl)ethenyl]benzene-1,3-diol and the molecular formula of Impurity-**B** is C_21_H_18_O_3_.

### Structural characterization of Impurity C

Impurity-C at RRT 3.42 was isolated by preparative HPLC. The LC-MS analysis of the fraction showed a molecular ion peak at m/z 317, which corresponds to the molecular weight of 316 as [M-H]^−^. The IR spectrum displayed characteristic absorptions at 3392 (broad signal), 3031, 2947, 1595, 1499, and 1460 cm^−1^ which is indicative of O-H, aromatic C-H, methylene C-H, and aromatic -C=C stretching. The structure was further supported by quaternary carbon signals in ^13^C and the DEPT spectrum. ^13^C NMR accounted for 20 carbons and the DEPT spectrum displayed only one negative carbon (CH_2_) signal and 14 positive carbon signals (12 aryl CH and 2 alkene CH) and the remaining six carbon signals appearing in ^13^C are considered as six quaternary carbons.

^1^H and ^13^C NMR results indicated that Impurity-C has five additional aromatic hydrogens/carbons and one aliphatic -CH_2_ compared to that of resveratrol. ^1^H NMR and D_2_O exchange studies indicated that this compound has only two hydroxyl groups compared to resveratrol which has three hydroxyl groups. The additional aliphatic -CH_2_- was deshielded to δ 5.07 ppm in ^1^H NMR and deshielded to δ 69.9 ppm in ^13^C NMR (Table 2). The above results indicate that the -CH_2_- group may be attached to oxygen which is more electronegative and hence supports the above NMR observation. As there is no significant change in ^1^H and ^13^C NMR chemical shift values of the phenyl ring having one hydroxyl group, it was concluded that the benzylic substitution may be present in the phenyl ring having two hydroxyl groups.

Based on the chemical shift deshielding of the hydrogen and carbon at the C6 position, the structure was arrived at with the benzyl substitution at the oxygen atom at the C5 position.

### Structural characterization of Impurity-D

Impurity-D at RRT 3.57 was isolated by preparative HPLC. The LC-MS analysis of the fraction showed a molecular ion peak at m/z 317, which corresponds to the molecular weight of 316 as [M-H]^−^. The IR spectrum displayed characteristic absorptions at 3392 (broad signal), 3032, 2924, 1603, 1512, and 1452 cm^−1^ which is indicative of O-H, aromatic C-H, methylene C-H, and aromatic –C=C stretching functionality. The structure was further supported by quaternary carbon signals in ^13^C and the DEPT spectrum. ^13^C NMR accounted for 20 carbons and the DEPT spectrum displayed one negative carbon (CH_2_) signal and 14 positive carbon signals (12 aryl CH and 2 alkene CH) and the remaining six extra signals appearing in ^13^C are considered as six quaternary carbons.

^1^H and ^13^C NMR results indicate that Impurity-D has five additional aromatic hydrogens/carbons and one additional aliphatic -CH_2_ compared to that of trans-resveratrol. ^1^H NMR and D_2_O exchange studies indicate that this compound has only two hydroxyl groups compared to resveratrol which has three hydroxyl groups. In ^1^H and ^13^C NMR, the additional aliphatic -CH_2_- was deshielded to δ 5.11 and δ 70.1 ppm, respectively.

The above results indicate that the -CH_2_- group may be attached to oxygen, which is more electronegative and hence supports the above observation. As there is no significant change in ^1^H and ^13^C NMR chemical shift values of the phenyl ring having two hydroxyl groups, it was concluded that the benzylic substitution may present in the phenyl ring having one hydroxyl group at the C12 position. The aromatic hydrogens at C-11 and C-13 were deshielded to δ 7.00 ppm from δ 6.74 ppm in the parent compound trans-resveratrol. Also, the hydrogens at C-10 and C-14 were also deshielded to δ 7.48 ppm from δ 7.38 ppm in trans-resveratrol (Table 2).

On the basis of the above spectral data, the structure of Impurity-D was assigned as 5-{(*E*)-2-[4-(Benzyloxy)phenyl]ethenyl}benzene-1,3-diol and the molecular formula of Impurity-**D** is C_21_H_18_O_3_.

### Structural characterization of Impurity-E

Impurity-E is the penultimate intermediate of trans-resveratrol. Though this impurity was not observed in the product, it was included in the impurity profile as per regulatory requirements. Impurity-E is 1,3-bis(benzyloxy)-5-{(*E*)-2-[4-(benzyloxy)phenyl]ethenyl}-benzene and molecular formula is C_35_H_30_O_3_.

## Conclusion

Information about the various possible impurities is a prerequisite for the thorough understanding of impurity profiles in the manufacturing of trans-resveratrol. Keeping in mind the regulatory requirement for impurities in trans-resveratrol, three process-related impurities were identified, isolated, and characterized by preparative HPLC, analytical HPLC, LCMS, IR, and NMR spectral data.

## Figures and Tables

**Tab. 1:** Impurity Profile of trans-resveratrol

Impurity	Retention time of the Impurity	Relative Retention time of the Impurity	Impurity Content (%)		

			TUXP050001	TUXP050002	TUXP050003
A	13.22*	1.339	–	–	–
B	29.91	3.029	0.10	0.06	0.06
C	33.79	3.422	0.42	0.36	0.20
D	35.29	3.574	–	0.12	0.04
E	56.98**	5.770	–	–	–

* Cis Resveratrol: Not observed in the samples.

** Penultimate Intermediate of Trans-resveratrol: Not observed in the samples.

**Tab. 2:** ^1^H and ^13^C NMR assignments for trans-resveratrol and Impurities A, B, C & D

Atom	Resveratrol				Impurity-A				Impurity-B				Impurity-C				Impurity-D			

	^1^H	ppm J	^13^C	DEPT	^1^H	ppm J	^13^C	DEPT	^1^H	ppm J	^13^C	DEPT	^1^H	ppm J	^13^C	DEPT	^1^H	ppm J	^13^C	DEPT
1			128.1				133.0				137.0				138.1				137.9	
2	1	6.37/d 2.2	104.4	CH	1	6.11/d 2.2	106.4	CH	1	6.41/s	105.0	CH	1	6.53/s	104.2	CH	1	6.38/s	105.3	CH
3			158.6				158.4				157.1				160.5				159.4	
4	1	6.10/t 2.2	101.9	CH	1	6.05/t 2.1	101.6	CH			114.4		1	6.28/t 1.9	101.9	CH	1	6.11/s	102.8	CH
5			158.6				158.4				157.1				158.2				158.8	
6	1	6.37/d 2.2	104.4	CH	1	6.11/d 2.2	106.4	CH	1	6.41/s	105.0	CH	1	6.68/s	106.9	CH	1	6.38/s	105.3	CH
7	1	6.79/d 16.2	125.7	CH	1	6.25/d 12.3	132.0	CH	1	6.73/s	126.0	CH	1	6.84/d 16.4	126.1	CH	1	6.86/d 16.4	127.6	CH
8	1	6.90/d 16.2	127.9	CH	1	6.35/d 12.3	132.1	CH	1	6.73/s	126.4	CH	1	7.02/d 16.4	128.6	CH	1	6.94/d 16.4	128.3	CH
9			139.4				139.2				142.7				140.3				139.9	
10	1	7.38/d 8.3	127.9	CH	1	7.06/d 6.7	131.5 131.6	CH	1	7.30/d 8.3	128.6	CH	1	7.45/d 8.5	128.5	CH	1	7.48/d 8.5	128.5	CH
11	1	6.74/d 8.3	115.6	CH	1	6.61/d 6.7	115.0	CH	1	6.66/d 8.4	116.4	CH	1	6.75/d 8.5	116.4	CH	1	7.00/d 8.5	115.9	CH
12			157.3				156.7				157.1				159.4				159.4	
13	1	6.74/d 8.3	115.6	CH	1	6.61/d 6.7	115.0	CH	1	6.66/d 8.4	116.4	CH	1	6.75/d 8.5	116.4	CH	1	7.00/d 8.5	115.9	CH
14	1	7.38/d 8.3	127.9	CH	1	7.06/d 6.7	131.5 131.6	CH	1	7.30/d 8.3	128.6	CH	1	7.45/d 8.5	128.5	CH	1	7.48/d 8.5	128.5	CH
15	1	9.30 bs			1	9.19 bs			2	3.75/s	27.4	CH_2_	2	5.07/s	69.9	CH_2_	2	5.11/s	70.1	CH_2_
16	1	9.30 bs			1	9.19 bs					129.3				129.3				130.7	
17	1	9.30 bs			1	9.19 bs			1	7.10–7.17/m	128.7	CH	1	7.38–7.42/m	128.8	CH	1	7.43–7.45/m	128.6	CH
18									1	7.10–7.17/m	129.3	CH	1	7.38–7.42/m	129.3	CH	1	7.36–7.40/m	129.3	CH
19									1	7.00/t 7.2	128.0	CH	1	7.31–7.35/m	129.3	CH	1	7.29–7.33/m	128.7	CH
20									1	7.10–7.17/m	129.3	CH	1	7.38–7.42/m	129.3	CH	1	7.36–7.40/m	129.3	CH
21									1	7.10–7.17/m	128.7	CH	1	7.38–7.42/m	128.8	CH	1	7.43–7.45/m	128.6	CH
22									1	9.33 bs			1	9.50 bs			1	9.20 bs		
23									1	9.33 bs			1	9.50 bs			1	9.20 bs		
24									1	9.33 bs										

Refer Fig. 2 for numbering;

s=singlet, bs=broad singlet, d=doublet, t=triplet, m=multiplet.
